# Is Malnutrition Associated with Crowding in Permanent Dentition?

**DOI:** 10.3390/ijerph7093531

**Published:** 2010-09-27

**Authors:** Erika B. A. F. Thomaz, Maria Cristina T. Cangussu, Antônio Augusto M. da Silva, Ana Marlúcia O. Assis

**Affiliations:** 1 Department of Public Health, Federal University of Maranhão, Maranhão, Rua Barão de Itapary, 155, Centro, São Luís, Maranhão, CEP: 65020–070, Brazil; E-Mail: aamouradasilva@gmail.com; 2 Dentistry School, Federal University of Bahia, Av. Araújo Pinho, 32, Canela, Salvador, Bahia, CEP: 40110–150, Brazil; E-Mail: cangussu@ufba.br; 3 Nutrition School, Federal University of Bahia, Av. Araújo Pinho, 32, Canela, Salvador, Bahia, CEP: 40110–150, Brazil; E-Mail: amos@ufba.br

**Keywords:** malocclusion, epidemiology, nutrition, protein-energy malnutrition

## Abstract

Evidence suggests that energy-protein malnutrition is associated with impaired growth and development of facial bones. The objective of this study was to investigate the association between nutritional status and reduced space for dental eruption (crowding) in permanent dentition. A cross-sectional study with probabilistic sampling design was used. We evaluated 2,060 students aged 12 to 15 years enrolled in schools in the northeast of Brazil. Crowding was defined according to World Health Organization (WHO) as misalignment of teeth due to lack of space for them to erupt in the correct position. Nutritional status was evaluated by means of body mass index and height-for-age, using the WHO’s reference curves. Parents and adolescents responded to a questionnaire about demographic, socioeconomic, biological and behavioral characteristics. The associations were estimated by odds ratio (OR) in multivariate logistic regression analysis (alpha = 0.05). Confounding and effect-modification were taken into account. An association between low height-for-age (z-score < −1SD) and crowding was only observed in adolescents with a prolonged history of mouth breathing (OR = 3.1). No association was observed between underweight and crowding. Malnutrition is related to crowding in permanent dentition among mouth-breathing adolescents. Policy actions aimed at reducing low height-for-age and unhealthy oral habits are strongly recommended. However, further studies are needed to increase the consistency of these findings and improve understanding of the subject.

## 1. Introduction

The relationship between nutritional status and oral health has recently become a subject of study [1–4]. Poor oral habits have traditionally been considered the main environmental factor responsible for poor dental occlusion [5]. However, some studies suggest that nutritional status may also be associated with malocclusion [4,6,7].

Malnutrition is a multifactorial disease that can have an early onset during intrauterine life or childhood or can occur during an individual’s lifetime as a result of poor nutrition and/or repeated episodes of infectious or chronic diseases [8]. The highest rates of energy-protein malnutrition (EPM) are recorded in developing countries [9] and represent a major nutritional problem, both because of their magnitude and the health disorders they give rise to [8,10]. EPM is associated with economic deprivation [11] and is an important indicator of a population’s quality of life [12].

Evidence suggests that energy-protein malnutrition acts by either exacerbating an existing morbidity or contributing to the emergence of associated comorbidities. In the field of oral health, the association between malnutrition and impaired growth and the development of facial bones has been reported by a number of researchers [6,13,14] and has been linked to a reduction in the length of the skull base and jaw height [15]. There have also been reports of variations in maxillomandibular width, lower facial height [13] and dental and skeletal ages [6,14,16,17] as a result of malnutrition.

It is believed therefore that malnutrition may also be associated with malocclusion, particularly dental crowding, which is defined as misalignment of the teeth due to insufficient space for them to erupt in the correct place [18]. Altered bone growth in the craniofacial complex caused by poor nutrition could be reflected in reduced space for dental eruption. There is evidence in animal models that support this hypothesis [19,20]. The authors evaluated the effects of dietary deficiencies of protein and calorie on the growth of jaws and teeth in pigs and observed that both the size and shape of the mandible were substantially affected. Moreover, the amount of space available for the teeth was reduced in the protein and calorie deficient animals (experimental groups) and crowding was substantially increased in the experimental animals [19,20]. Another study, in rats, also found reduction in the size of the jaw in animals with protein and calorie malnutrition [21]. These findings suggest that malnutrition produces relative and absolute changes in the spatial arrangements of the teeth in the jaws. To our knowledge, there is only one study in humans that investigated the relationship between underweight or low height-for-age and dental crowding in the literature; however, the study only included low-income children with deciduous teeth [4]. The authors, who studied children aged 3 to 5 years, found that low weight for age was associated with a high risk of dental crowding but failed to find an association between the latter and low height for age. The aim of the present study was therefore to investigate, for the first time, the relationship between anthropometric failure and dental crowding in a large sample of adolescents in permanent dentition.

## 2. Materials and Methods

### 2.1. Study Type and Sample Design

The study was a cross-sectional population-based study involving 12 to 15-year-old adolescents in the city of Salvador, BA, Brazil. It was estimated that a sample of 1,580 individuals would have a 80% chance of detecting a 10% difference in the prevalence of dental crowding—estimated at around 35% [22]—between exposed and unexposed groups, with α = 0.05, considering a design effect of two. However, to compensate for possible losses and missing data and to detect possible interactions, the study sample was increased by 30%, to 2,060.

Probabilistic two-stage cluster sampling was used. The schools were the primary sampling units and the adolescents, the secondary units. The sample was further stratified into two groups according to whether the school was a private or public one. There were 398 primary schools in the city and 196,532 students enrolled (83.4% in public schools and 16.6% in private ones) [23]. The same population proportions were maintained in the sample. Forty schools (33 public and seven private), representing 10% of the total, were chosen at random; one additional private school was added later to make up the minimum number of students per school because one of the private schools chosen did not have enough eligible students. To ensure that all the age groups of interest were represented in the study sample, 13 adolescents were selected in each age group, yielding a total of 52 adolescents from each school.

If the student selected was not found in the school at the time of the assessment, the team returned to the school on two additional occasions, after which the information relating to that adolescent was considered a loss.

### 2.2. Inclusion and Exclusion Criteria

Students aged 12 to 15 years enrolled in the 6th to 9th grades of public or private secondary schools in the municipality of Salvador who had permanent teeth were included in the study. The following individuals were excluded: those who refused to submit to the evaluations (n = 2); those whose parents or guardians did not sign the written voluntary informed-consent form (n = 187); pregnant women (n = 1); adolescents with a physical handicap or who were unable to move because of physical trauma, as anthropometric measurements could not have been taken (n = 3); and those who reported prior or current use of an orthodontic or orthopedic appliance (n = 4).

### 2.3. Definition of the Variables and Data Collection

Dental crowding was defined according to the World Health Organization (WHO) [18] as a misalignment in the teeth position (in millimeters), using the WHO periodontal probe. It was the dependent variable and, to these analyses, it was classified as present (in one or both arcades) or absent (in neither arcade).

There were two main independent variables assessing the nutritional status of adolescents: nutritional status according to the BMI and height-for-age indexes. The authors acknowledge that assessment of the nutritional status of adolescents is made difficult by variations in growth, by the extent to which the adolescents had grown in previous periods of life and by the intricate relationship between sexual maturation and hormonal factors during this period of life [24]. Nevertheless, the WHO recommends the body mass index (BMI) and height-for-age index as suitable indicators for evaluating the nutritional status of adolescents, and these were used in this study.

For both indicators, the reference curves and cut-off points recently advocated by the WHO for adolescents and children above the age of five years were adopted [25,26]. The following classification was used: severe thinness (BMI z-score of less than −3SD); thinness (−3SD ≤ BMI z-score < −2SD); risk of thinness (−2SD ≤ BMI z-score < −1SD); normal BMI-for-age (−1SD ≤ BMI z-score ≤ +1SD); and overweight (+1SD < BMI z-score ≤ +2SD). Obesity is classified as +2SD < BMI ≤ +3SD, and severe obesity as BMI z-score > +3DP [26]. In the present study, the following categories were defined for BMI: normal BMI-for-age/risk of thinness (−2SD ≤ BMI z-score ≤ +1SD), thinness/severe thinness (BMI z-score < −2SD); and overweight/obesity (BMI z-score > +1SD) [25,26].

A height-for-age deficit was considered moderate/severe when the z-score was more than 2SD below the median for the reference population and mild when −2SD ≤ z-score < −1SD. A z-score ≥ −1SD was considered to indicate normal/high/very high height-for-age [25,26]. Because of the low prevalence of stunting in the sample (4.58%, n = 88), it was decided to group all types of malnutrition [as measured by the height-for-age index] together in a single category, and the adolescents were classified as having normal height-for-age (z-score ≥ −1SD) or some degree of low height-for-age (mild/moderate/severe: z-score < −1SD).

The co-variables in the study, measured by using a questionnaire answered by the students and their mothers/guardians, were: (a) demographic—age in years (*‘12–13’* or *‘14–15’*), sex (*‘male’* or *‘female’*), color (*‘white’, ‘brown’* or *‘black’*); (b) behavioral—*duration of* nutritious sucking habits (natural and artificial breast-feeding, categorized as *‘never breast-fed/breast-fed up to 12 months’* or *‘breast-fed for more than 12 months’*) and non-nutritious sucking (sucking a digit or pacifier), as well as poor oral habits such as nail-biting, bruxism (grinding/clenching teeth) and mouth breathing, both classified as ‘*never had the habit/had the habit but stopped at 6 years of age’* or *‘had the habit until after the age of six years’*; (c) biological—the absence of posterior teeth (*‘yes’* or *‘no’*); and (d) socioeconomic—the level of schooling of the head of the family (‘*high’*—higher-level education not completed to postgraduate qualification, ‘*average’*—primary and secondary education completed, or ‘*low’*—illiterate and primary education not completed), family income (‘*high’*—more than 5 minimum monthly wages, ‘*average’*—from 2 to 5 minimum monthly wages, or ‘*low’*—less than 2 minimum monthly wages) and the type of school in which the adolescent was studying (*public* or *private*). Color was defined according to the criteria of the Brazilian Geographic and Statistical Institute (IBGE) [27], and because of the small number of individuals with yellow skin coloration (Orientals) or indigenous natives in the study population, these were excluded from the study (n = 19).

Only those interviewers/examiners with concordances equal to or greater than 0.85 were selected for the team. Occlusion was evaluated according to the WHO recommendations [18], and the anthropometric measurements followed Lohman *et al.* [28]. Duplicate measurements were taken in a double-blind test, and an intra-measurer variability of 100 g for weight and 0.1 cm for height were considered acceptable. The mean of two measurements was used as the final measurement.

### 2.4. Data Analysis

The data were entered into the database in duplicate. The AnthroPlus^®^ package [26] was used for the anthropometric analysis, and Stata/SE^®^ version 9.0 was used for the remaining statistical analysis.

Descriptive analysis and logistic regression were carried out. Odds ratio (OR) and respective 95% confidence intervals (95% CI) were estimated to investigate the associations of interest. The co-variables included in multivariate (adjusted) models were chosen based on statistical significance (alpha < 0.2) of the univariate (unadjusted) associations between dental crowding and the co-variables, as well as by means of theoretical criteria based on the currently available literature.

In the multivariate analysis, the likelihood ratio test (lrtest) was used to identify potential effect-modifying variables by comparing the unrestricted (model without product terms) and restricted models (model with first order product terms) (alpha = 0.05). For the analysis of confounding variables, the stepwise backward strategy was used to remove variables from the model, keeping those variables (considered confounding variables) that, when removed, produced a difference in individual measurements of the association between nutritional state and malocclusion equal to or greater than 10% [29]. Two different multivariate analyses were performed: one for BMI and another for the height-for-age index.

Estimates took into account the complex selection of the study sample. Standard errors were corrected, design effect (DEFF) was estimated, and the stratification variable and the variable representing the primary sample units were incorporated in the analysis so that the intra-cluster correlation was taken into account [30]. In addition, as the selection probability was not the same for adolescents of different ages and also depended on the school in which they were enrolled—students in smaller schools had a greater probability of being selected—the estimates were weighted by the inverse of the selection probability for each adolescent [30]. The variables age and school were used for the weighting.

## 3. Results

Three private-sector schools (7.5%) refused to take part in the study and were replaced by other schools selected at random. The return rate for questionnaires sent to adolescents was 100%, and for those sent to parents/guardians, 78.12%. Questionnaires that were not filled out completely represented another source of missing information.

There was a greater prevalence of dental crowding among adolescents with high BMI than among adolescents with normal BMI-for-age (OR = 0.66; 95%CI: 0.45–0.96). No variation in the distribution of dental crowding with nutritional status as measured by height-for-age was observed (OR = 1.13; 95%CI: 0.89–1.42). In addition, bottle feeding for more than one year acted as a statistically significant potential risk factor for dental crowding (OR = 1.42). The following, however, were considered potential protection factors: coming from a low-income family (OR = 0.33), studying at a public school (OR = 0.27) and having a history of breast-feeding for more than 12 months (OR = 0.66) (Table 1).

Multivariate analysis of the association between nutritional status, as measured by BMI, and the presence of dental crowding failed to reveal an association after an adjustment had been made for confounding variables (Table 2). Breast-feeding and digit sucking were kept in the multivariate models even without showing statistical significance in the multivariate model due to theoretical reasons.

There was an interaction between mouth breathing and malnutrition, as measured by the anthropometric height-for-age index. So the effect of malnutrition on dental crowding was modified by mouth breathing (p-value in the lrtest = 0.04–data not shown). After adjustment for confounding variables, a statistically significant association between low height-for-age and dental crowding (OR = 3.1; 95%CI: 1.56–6.17) was only observed among adolescents with a history of mouth breathing for a longer time (until the age of 6 or longer) (Table 3).

## 4. Discussion

We failed to find an association between underweight and crowding in permanent dentition. However, we did observe an association between low height-for-age and this orthodontic problem. This association was only observed among those that had breathed through the mouth for a long period, even after adjustment for family income, skin color, duration of breast-feeding, duration of bottle-feeding and a history of digit sucking.

There is some evidence in animal models that support the hypothesis of association between malnutrition and malocclusion using pigs [19,20] and rats [21]. The authors observed that dietary deficiencies of protein and calorie have effects on the reduction of the growth of jaws and of the space available for the teeth in these animals, resulting on increase of crowding in the experimental animals [19,20]. It may indicate that malnutrition changes the growth pattern of the bones of the skeleton, including those of the face and oral cavity.

To our knowledge no studies investigating this relationship in adolescents or adults have been published to date in humans. Only one study has been carried out, and this found an association between underweight and an increased chance of dental crowding in children aged 3 to 5 years with deciduous teeth but failed to find a relationship with low height-for-age [4]. We believe that the divergence between these findings can be explained primarily by the fact that the study subjects belonged to different age groups and were therefore exposed to nutritional deprivation for different lengths of time at different periods during craniofacial growth and development. Thus, children up to 5 years of age may not have been exposed to nutritional deprivation long enough for this to be reflected in maxillomandibular growth, as peak growth in these bone structures occurs at around 6 to 14 years of age in girls and 12 to 18 years in boys [31]. Consequently, the effects of mandibular or maxillary growth deficiency on dental crowding cannot be satisfactorily evaluated before the age of five.

Another plausible explanation is that the sample in the previous study was limited to children in public schools, who are worst off socioeconomically, making it impossible to provide a suitable contrast for this variable, which is considered important in predicting oral health [32]. Accordingly, when the estimates for the variables related to socioeconomic factors were adjusted in the present study, confounding was observed, and the association between underweight and crowding disappeared and the association between low height-for-age and dental crowding was revealed instead. This result agrees with the study hypothesis, as BMI alone is unsuitable for evaluating the chronic consequences of malnutrition in oral health [33], whereas the indicator height-for-age reflects the individual’s prior health and any diseases they have contracted and is thus a better marker for poor living standards, repeated infections during childhood and chronic food deprivation over longer periods of time

Irrespective of these divergences, the results of the present study show the association between low height-for-age and dental crowding and confirm the deleterious effects of mouth breathing on teeth occlusion, as also reported by other researchers [5,34]. Nutritional status has been associated with various oral health problems [1–3,10,35,36]. To our knowledge, however, this is the first study to find an association between linear deficit and dental crowding.

The mechanisms that might explain this relationship have yet to be fully clarified. One line of reasoning is based on the restricted growth and development of bones in general (and of the bones of the face in particular) in the presence of malnutrition accompanied by stunting. While this relationship has been suggested by a number of investigators, their conclusions are based on studies using small samples, for which the details of the methodology used were not described in full, preventing a more detailed analysis of the results. These studies suggest that stunting could be reflected in, for example, the height of the mandible [15], the height of the lower face and dental and skeletal ages [6,14,16,17, 37].

It is thus reasonable to hypothesize that low height-for-age can be associated with the restricted growth/development of the bones of the face and can change the amount of space available for permanent teeth to erupt in, rendering the association observed in this study more biologically plausible, as shown in animal studies [19–21].

This association also highlights the fact that oral habits modify the effect of malnutrition on dental crowding. The interaction found is plausible since both mouth breathing and malnutrition theoretically have the potential to affect the pattern of bone growth. Perhaps the chronic malnutrition renders the skeleton more susceptible to the action of other environmental factors. Mouth breathing is one of the local factors that have a more adverse effect on dental occlusion [5], insofar as the absence of the flow of air through the nasal cavity and the muscular imbalance brought about by breathing through the mouth can result in insufficient vertical growth of the maxilla and mandible [34,38] as well as inadequate transverse growth in these bones [39], which is reflected in a lack of space for teeth to erupt in.

It is also possible that crowding may be indirectly related to nutritional status by means of impaired odontogenesis, leading to delayed dental eruption [6,14,16,40] and an increased incidence of caries [36,37], with a consequent loss of teeth. Such events could compromise the shape of the dental arch [41] and be reflected in maxillomandibular growth in the form of insufficient masticatory stimulus. Individuals who lose their deciduous teeth early because of caries could be more susceptible to tooth migration and occlusion problems, such as dental crowding [42]. The loss of permanent teeth, in contrast, could have the opposite effect, increasing the length of alveolar ridge available for teeth to occupy, thereby reducing crowding [43]. As premature loss of these dental elements usually occurs among populations with a low income and poor level of education [44], dental crowding can be expected to have a greater prevalence in adolescents whose families are financially better off, as observed in this study.

This reasoning is in line with the functional matrix hypothesis [45], according to which the face grows in response to functional needs throughout an individual’s life. The shape of the dental arch would thus be strongly influenced by oral functions, by vertical growth of the alveolar processes in response to the stimulus of dental eruption [41] and by the muscular pressure exerted on these tissues, whereas the loss of teeth would adversely affect this process, highlighting the importance of environmental factors in the arrangement of teeth and the relationship between the bones in the maxillomandibular complex.

The study has a number of strengths: its pioneering nature; the biological plausibility of the association observed; the use of diagnostic criteria for malocclusion validated and recommended by international bodies; the use of anthropometric indicators reflecting both current body composition and events since the very early period of an individual’s life that limited physical growth; the carefully performed statistical analysis; and the accuracy of the estimates. However, some drawbacks make caution necessary when analyzing our results.

Despite being suitable for investigating associations between variables, the cross-sectional design used here is unsuitable for inferring causality and is prone to recall bias when individuals are attempting to remember prior exposures. However, this may have not been an important study limitation since both explanatory and response variables were measured objectively. However, recall bias may have affected some confounding variables. Nevertheless, by using the height-for-age indicator the supposition that there has been prior exposure holds, even in a cross-sectional study. In addition, the use of relatively broad cut-off points for some of the main co-variables helps to reduce the margin of error during classification as a result of memory bias. Furthermore, in an attempt to quantify this error, information was collected from two different sources: the student and the mother/guardian. The concordance (*kappa*) between the information from these two sources was considered good and varied from 90.72% (nail-biting) to 99.37% (level of education of the head of the family).

Despite the use of self-completed questionnaires in an attempt to reduce embarrassment and minimize errors due to incorrect answers or unanswered questions, especially in relation to oral habits and socioeconomic conditions, our efforts may not have been completely successful, as these variables had the highest rates of unanswered questions, particularly among adolescents in private schools, and may have led to biased estimates.

The variable mouth breathing (presence and duration) was defined based on reports from parents/guardians and not by experts (the gold standard criterion). So, it was subject to classification bias. However, some recent studies have validated and recommended the use of self-reported morbidity as an important diagnostic tool in epidemiological studies [46–48]. Analysis of the validity of this information, when compared to other methods, has shown high specificity and satisfactory sensitivity and kappa [48]. Moveover, even using a subjective criterion, it was possible to record the deleterious effects of mouth breathing in the occurrence of malocclusion (crowding), which is consistent with other studies that point out the deleterious effects of mouth breathing in the growth and development of facial structures [5,34,38,48].

Lastly, the theoretical model used in this study proved to have little explanatory value, suggesting that other variables, such as genetic inheritance, need to be included in future multivariate analyses. So, considering that the dental crowding is one of the most important malocclusions that predicts the orthodontic treatment need, especially due to its impact on the facial aesthetic, the study of the factors associated with the high prevalence of dental crowding have contribution to health public.

## 5. Conclusions

It is reasonable to suggest that poor health and nutrition are related to crowding in permanent dentition among mouth-breathing adolescents. Therefore, policy actions aimed at reducing low height-for-age and unhealthy oral habits are strongly recommended in order to reduce malocclusion. However, due to the limitations of this study, further studies are needed to increase the consistency of these findings and improve understanding of the subject.

## Figures and Tables

**Table 1.** Frequency distribution and univariate (unadjusted) analysis of the association between dental crowding and demographic, social, economic and behavioral characteristics among adolescents. Brazil, 2004.

**Variables**	**Dental crowding**							

	**No**		**Yes**		**p-value**	**DEFF**	OR***	**95%CI**
	**n**	**%**	**n**	**%**				
BMI					0.03*	1.21		
Normal BMI-for-age	780	77.5	711	79.9	0.66**		0.66	0.45–0.96
Overweight/Obesity	155	14.9	110	10.0			1.00	--
Underweight	73	7.6	86	10.1			1.28	0.84–1.95
No information^1^	77	--	68	--				
Height-for-age					0.30*	1.21		
Tall/normal	774	76.0	674	73.8			1.00	--
Malnourished	240	24.0	233	26.2			1.13	0.89–1.42
No information^1^	71	--	68	--				
Age					0.29*	1.24		
12–13 years	578	53.1	492	50.5			1.00	--
14–15 years	507	46.9	483	49.5			1.11	0.91–1.35
Sex					0.68*	1.64		
Male	461	43.6	431	44.7			1.00	--
Female	624	56.4	544	55.3			0.95	0.76–1.20
Color					0.13*	2.24		
White	42	3.7	62	4.9	<0.001**		1.00	--
Brown	741	65.5	702	70.2			0.82	0.43–1.54
Black	293	30.8	205	24.9			0.62	0.34–1.13
No information^1^	9	--	6	--				
Family income^2^					0.07*	1.20		
+ 5 m. monthly wages	32	2.1	66	4.5	<0.001**		1.00	--
2–5 m. monthly wages	203	26.0	207	25.3			0.45	0.21–0.99
<2 m. monthly wages	519	71.9	451	70.2			0.45	0.23–0.91
No information^1^	331	--	251	--				
Level of Education^3^					0.53*	1.56		
High	30	2.3	53	3.4	0.02**		1.00	--
Average	384	48.2	366	47.0			0.66	0.30–1.42
Low	379	49.5	330	49.6			0.33	0.30–1.52
No information^1^	297	--	226	--				
Breast-feeding					0.53*	2.56		
Never-up to 12 months	613	72.5	584	74.7			1.00	--
More than 12 months	218	27.5	187	25.3			0.66	0.47–0.93
No information^1^	254	--	204	--				
Bottle feeding					0.02*	0.99		
Never-up to 12 months	174	17.9	101	13.3			1.00	--
More than 12 months	685	82.1	669	86.7			1.42	1.06–1.92
No information^1^	253	--	205	--				
Posterior teeth lost					0.61*	1.13		
No	940	85,4	849	86.3			1.00	--
Yes	145	14,6	126	13.7			0.93	0.71–1.23
Mouth breathing					0.41*	1.24		
Never- up to 6 years	510	63,8	456	61.5			1.00	--
More than 6 years	298	36,2	290	38.5			1.10	0.87–1.40
No information^1^	277	--	229	--				
Digit sucking					0.80*	1.71		
Never- up to 6 months	728	87,5	662	86.9			1.00	--
>6 years	104	12,5	110	13.1			1.05	0.71–1.56
No information^1^	253	--	203	--				
Pacifier sucking					0.19*	2.63		
Never- up to 6 months	779	93.9	710	91.5			1.00	--
More than 6 years	51	6.1	56	8.5			1,43	0.81–2.51
No information^1^	255	--	209	--				
Bruxism					0.27*	1.76		
Never- up to 6 months	614	73.9	585	77.2			1.00	--
More than 6 years	203	26.1	177	22.8			0.84	0.61–1.16
No information 1	268	--	213	--				

Weighted estimates for the design effect (DEFF) and for the inverse of the selection probability for the subjects. The prevalence of dental crowding was 47.7% (n=975; 95% CI: 42.0–53.0).

BMI = Body Mass Index; OR = odds ratio; 95% CI = 95% confidence interval.

1 Incomplete or blank responses were not taken into consideration in the statistical analysis.

2 Minimum monthly wage was R$ 260.00 (U$ 90.1) during the period August to October 2004.

3 Level of education: high (higher-level education not completed/postgraduate qualification), average (primary education completed/secondary education completed), low (illiterate/primary education not completed).

* Pearson chi-square test.

** Chi-square test for trend.

*** Unadjusted Odds Ratio.

**Table 2.** Odds ratio and the respective 95% confidence intervals for the association between dental crowding and nutritional status (according to Body Mass Index) and family income. Brazil, 2004.

	**OR**	**95%CI**	**p-value**
BMI			
Overweight/Obesity	0.85^**^	0.44–1.67	0.64
Normal BMI-for-age	1.00	--	--
Underweight	1.36^**^	0.59–3.15	0.45
Family income^*^			
+ 5 minimum monthly wages	1.00	--	--
2–5 minimum monthly wages	0.42^***^	0.16–1.14	0.08
<2 minimum monthly wages	0.35^***^	0.14–0.94	0.03

BMI = Body Mass Index; OR = odds ratio; 95%CI = 95% confidence interval.

* Minimum monthly wage was R$ 260.00 (U$ 90.1) during the period August to October 2004.

** Adjusted for family income, color, duration of breast-feeding and bottle feeding, digit sucking and pacifier sucking.

*** Adjusted for BMI, color, duration of breast-feeding and bottle feeding, digit sucking and pacifier sucking.

**Table 3.** Odds ratio for dental crowding and the respective 95% confidence intervals for combined nutritional status (according to height-for-age) and history of mouth breathing. Brazil, 2004.

	OR*	**95%CI**	**p-value**
No history of mouth breathing or a history of mouth breathing up to 6 years of age.	1.28	0.78–2.09	0.32
A history of mouth breathing until after 6 years of age.	3.10	1.56–6.09	<0.001

OR = odds ratio; 95% CI = 95% confidence interval.

* Adjusted for color, family income, duration of breast-feeding and bottle feeding, history of digit sucking and pacifier sucking.
